# Easing the Burden of Tinnitus: A Narrative Review for Exploring Effective Pharmacological Strategies

**DOI:** 10.7759/cureus.54861

**Published:** 2024-02-25

**Authors:** Seung Ho Kim, Ikhee Kim, Hantai Kim

**Affiliations:** 1 Department of Otorhinolaryngology-Head and Neck Surgery, Konyang University College of Medicine, Daejeon, KOR

**Keywords:** intratympanic injection, carbamazepine, anxiolytics, antidepressants, medical treatment, tinnitus

## Abstract

Many individuals seek medical attention for tinnitus, desiring relief from the distress caused by the condition; however, the treatment process is far from straightforward. The most effective treatments for chronic subjective tinnitus, such as tinnitus retraining therapy (TRT) and cognitive behavioral therapy (CBT), require considerable time and efforts. As a result, many of them express a desire for alleviation through medication. While it is true that medication is not generally recommended in treatment guidelines for chronic subjective tinnitus, in specific situations such as when accompanied by symptoms of depression or anxiety-drugs like antidepressants or anxiolytics may have a meaningful impact on symptom reduction. Additionally, medication can prove effective in certain specialized forms of tinnitus, such as typewriter tinnitus, as opposed to chronic subjective tinnitus. Although intratympanic dexamethasone injections for tinnitus have been reported to lack efficacy compared to a placebo, if patients perceive subjective satisfaction due to a placebo effect, it holds significance. From the perspective of patients suffering from tinnitus, even if the therapeutic mechanism is set aside, experiencing some degree of relief through certain medications can enhance compliance with evidence-based treatments like TRT and CBT.

## Introduction and background

The perception of sounds in the ears or head without any actual external sound is referred to as tinnitus. Reports suggest that the prevalence of experiencing tinnitus at least once ranges from 5% to 30%, indicating its common occurrence [1-5]. It is even more prevalent in the elderly, with approximately one in three individuals experiencing tinnitus [6,7]. While experiencing tinnitus on a singular basis might not warrant significant concern, it is noteworthy that 1-7% of patients report tinnitus with a level of distress significant enough to impact their daily lives [3-5,8]. Consequently, tinnitus, presenting as a relatively common condition in otolaryngology clinics, is gaining prominence, especially when it causes substantial discomfort affecting the quality of life.

Current clinical guidelines for tinnitus

There are several guidelines for the treatment of tinnitus. The 2014 guidelines in the United States recommend “Education and counseling,” “Cognitive behavioral therapy (CBT),” and “Hearing aid evaluation” [9]. Tinnitus retraining therapy (TRT) is not explicitly addressed separately, as TRT consists of directive counseling, sound therapy, and the use of hearing aids for hearing loss [10]. Each of these components can be considered individually. Sound therapy, a component of TRT, is not specifically endorsed and is considered more of an optional measure. On the other hand, medical therapy goes beyond being not recommended; it leans toward advising against its use. The guidelines discourage the routine use of antidepressants, anticonvulsants, anxiolytics, and intratympanic drug administration for tinnitus treatment, emphasizing the need to avoid their routine usage [9].

The 2019 European guidelines strongly recommend CBT [11]. However, there is no specific endorsement (no recommendation) for TRT, primarily due to a lack of high-level research providing clear evidence of its effectiveness. In cases of severe hearing loss, cochlear implantation (CI) is recommended, while the use of hearing aids, when feasible, is recommended for managing tinnitus associated with hearing loss. The guidelines also maintain a negative stance on medications. They advise against the use of antidepressants in patients without a diagnosed depressive disorder and emphasize that anxiolytics, while helpful in managing accompanying anxiety, do not impact tinnitus itself [11].

The Japanese guidelines released in 2020 also acknowledge the cost-effectiveness of CBT [12]. The use of hearing aids and sound therapy, grouped under TRT, is considered effective as well. While the guidelines do not outright oppose the use of medications, they highlight the difficulty in predicting their effectiveness and suggest that medications should be used specifically to reduce accompanying depression or anxiety [12]. Finally, a brief overview of the most recent German guidelines published in 2022 reveals that on the first page, counseling, psychotherapeutic intervention, and measures to improve hearing are identified as the most effective therapeutic interventions. Other treatment methods, including medications, are stated to be either ineffective or lacking sufficient evidence [13]. Several studies that have analyzed these guidelines in detail also show a similar overall trend [14,15].

In summary, it can be considered that only CBT and TRT (which includes counseling and hearing aid usage) have clear evidence as effective treatments. Unfortunately, the use of medications is found to have very limited supporting evidence, despite the fact that they are frequently prescribed for the purpose of treating tinnitus.

Consequently, tinnitus, presenting as a relatively common condition in otolaryngology clinics, is gaining prominence, especially when it causes substantial discomfort affecting the quality of life.

Considerations for medication-based interventions

Despite guidelines and clinical recommendations advocating against the use of medications for tinnitus treatment, prescribing drugs for tinnitus management remains prevalent in clinical settings. According to a study, medication prescriptions constitute the highest proportion of treatments for tinnitus, with 45.4% of all tinnitus patients receiving medication, while CBT, considered most effective, is prescribed to only 0.2%. TRT accounts for just 3%, and surprisingly, nutritional supplements rank third at 7.8%, making drug prescriptions represent more than half of the treatment approaches [3]. This indicates a deviation from guideline-based practices, emphasizing the need to consider the preferences and needs of tinnitus patients.

A survey revealed that when asked about their expectations regarding tinnitus treatment methods (allowing multiple responses), 36.5% responded with “I have no expectation,” while medication usage had the next highest rate at 28.7%. Expectations for the use of vitamins or supplements were also significant at 24.3% [16]. Despite guidelines restricting the use of medications, it is evident that many patients anticipate symptom improvement through drug intake. This inclination is understandable, as taking medication is generally more straightforward. TRT or CBT may require a considerable amount of time, and considering the potential costs in the treatment, it is understandable that patients might favor the simplicity of medication. For physicians encountering tinnitus patients, garnering interest in non-pharmacological approaches proves challenging. Consequently, despite the limited universal efficacy of medications, devising effective strategies for medication use in tinnitus remains an unavoidable challenge.

Decoding tinnitus guidelines: Effective pharmacological treatments on patient perspectives

Pharmacological treatment can indeed have some efficacy in managing tinnitus. Upon careful examination of tinnitus treatment guidelines, one may find that pharmacological interventions can be surprisingly effective in specific cases. It is worth noting that the guidelines discussed primarily target “primary chronic subjective tinnitus.” Stated differently, when tinnitus is not chronic subjective but, for instance, secondary to another condition, pharmacological treatments can occasionally prove quite effective. Consequently, when a physician encounters a patient with tinnitus resulting from a secondary cause, adjusting the primary cause (especially if it is modifiable through medication) can effectively control tinnitus. From a medical practitioner's perspective, secondary tinnitus of this nature might not be considered a strictly primary form of tinnitus. However, patients who undergo treatment may perceive the pharmacological intervention as effectively treating their tinnitus.

Another aspect to consider is the placebo effect. Guidelines and various systematic reviews often indicate that there is no significant effect compared to a placebo. In some medications, the placebo effect can be quite strong, making it challenging to demonstrate the actual efficacy of the drug. However, from the patient's perspective, whether it is a placebo effect, a genuine effect of the medication, or due to some underlying mechanism might not be as crucial. What matters most is the reduction in the personal distress caused by their tinnitus. Physicians treating tinnitus patients can, in fact, appropriately leverage these placebo effects when meeting and managing patients.

Whether it is the modulation of accompanying symptoms or driven by a placebo effect, if patients can experience even a slight reduction in the distress caused by their tinnitus, it is challenging to deny the potentially positive impact on the responsiveness to more definitive treatments like TRT or CBT. In the subsequent detailed discussions on each medication, we aim to revisit various pharmacological treatments that have been proposed thus far, drawing on these considerations.

## Review

Tinnitus accompanied by anxiety and depression

A notable anxiolytic, benzodiazepine, specifically binds to the gamma-aminobutyric acid (GABA) receptors, facilitating the activity of the GABA neurotransmitter. Through this process, benzodiazepines effectively control symptoms of anxiety and depression [17]. The effectiveness of this medication in regulating anxiety symptoms is undisputed. However, caution is necessary due to the risk of developing drug dependency and potential abuse issues when used over an extended period [18]. Randomized controlled trials have been conducted with diazepam, alprazolam, oxazepam, and clonazepam to demonstrate their effectiveness in tinnitus [19-24]. When comparing the results, clonazepam appears to show relatively better efficacy in suppressing tinnitus compared to other medications. Clonazepam's longer half-life, compared to other drugs, reduces the risk of abuse, making it advantageous in terms of usage [18].

Research has been conducted on the tinnitus-inhibiting effects of tricyclic antidepressants (TCAs) and selective serotonin reuptake inhibitors (SSRIs) in the context of antidepressant use [25-32]. A Cochrane review published in 2012, which focused on four TCA studies and two SSRI studies, noted that both classes of antidepressants demonstrated that the evidence for or against their efficacy was not conclusive [33]. However, it is worth considering that SSRIs are generally perceived as more tolerable compared to TCAs or other monoamine oxidase inhibitors (MAOIs) [34,35]. If the use of antidepressants is deemed necessary, opting for SSRIs over TCAs would be more appropriate. In the previously mentioned Cochrane review, studies on paroxetine and trazodone among SSRIs showed no efficacy. However, a study targeting sertraline yielded positive results in tinnitus inhibition. In a study conducted by Zöger et al., where sertraline was administered (25 mg/day in the first week, then 50 mg/day for a total of 16 weeks), the Tinnitus Severity Questionnaire (TSQ) significantly decreased compared to the control group. Additionally, the tinnitus loudness also decreased, suggesting effectiveness, especially in severe refractory tinnitus [31]. Nevertheless, the high dropout rate (17%) and the need to consider the correlation between the reduction in tinnitus and the decrease in depressive and anxious symptoms should be taken into account.

In fact, there are studies reporting a reduction in tinnitus when using anxiolytics or antidepressants. However, the observed effects are generally attributed to the benefits obtained from regulating concurrent depressive or anxious symptoms [36]. Consequently, while most tinnitus clinical guidelines express opposition to the routine use of such medications, they acknowledge the potential use of anxiolytics or antidepressants in cases where there is concurrent depression or anxiety [9,12,13]. From the perspective of tinnitus patients, the sounds perceived in the ear and the resulting anxiety or depression are often perceived as a unified burden. In some cases, anxiety or depression may even be the predominant issue. Therefore, patients of this nature may feel a reduction in distress caused by tinnitus when there is a decrease in concurrent anxiety or depression. Comprehensive interviews and, if necessary, brief surveys on depression and anxiety could be conducted. If the need for regulation of depression and anxiety is identified, the judicious use of appropriate medications may alleviate a significant part of the burden associated with tinnitus for the patient. This, in turn, could lead to a more positive therapeutic experience for the patient, potentially improving compliance with more ultimate treatments such as CBT or TRT.

Typewriter tinnitus

Certain atypical forms of tinnitus respond well to drug therapy, requiring a distinct approach from the clinical guidelines mentioned earlier, as they are not encompassed within chronic subjective tinnitus. However, patients with these types of tinnitus typically present with a chief complaint of perceiving sounds in the ear, just like other forms of tinnitus. Accurate patient interviews are crucial to identifying and appropriately treating such types of tinnitus. One notable example is typewriter tinnitus, considered a rare subtype of tinnitus. It manifests as a staccato-type sound, resembling the distinct, rhythmic tapping of a typewriter or the popping sound of corn. It can be triggered by head movements or specific sounds. This condition is known to occur when the eighth cranial nerve is compressed by the loop of the anterior-inferior cerebellar artery (AICA) [37].

The responsiveness to carbamazepine was initially reported in this regard [37], and through subsequent studies, there seems to be reasonably sufficient evidence to support this [38,39]. However, in clinical settings, accurately diagnosing this condition can sometimes be challenging. While the distinctive nature of typewriter tinnitus may make it seemingly straightforward to differentiate, it appears that many patients struggle to articulate it accurately. Conducting an MRI to examine neurovascular compression of the cochlear nerve could be considered for diagnosis [40]. On the other hand, some suggest that administering carbamazepine and assessing immediate responses to short-term treatment could hold diagnostic value [38]. Since immediate responses often occur within two weeks of commencing low-dose (200-400 mg daily) carbamazepine treatment [37,38,41], initiating carbamazepine could be a viable option when typewriter tinnitus is suspected, even if certain aspects of the diagnosis are slightly uncertain. It is important to note that symptoms may reappear upon discontinuation of the medication, and while existing literature suggests use for up to three months, additional research is warranted for cases requiring prolonged administration [38,39].

Especially when patients' descriptions of tinnitus sounds are somewhat uncertain, initiating carbamazepine can be helpful in cases where distinguishing between tinnitus due to middle ear myoclonus or pulsatile tinnitus, which can be challenging at times. If there is no response, considerations for tinnitus resulting from middle ear myoclonus or pulsatile tinnitus may arise. In cases of tinnitus due to middle ear myoclonus, conservative treatments involving muscle relaxants like baclofen or clonazepam are viable options [39,42]. However, in cases that are not effectively managed (intractable), surgical interventions such as the excision of the stapedial tendon or tensor tympani tendon may be more effective [43,44]. Pulsatile tinnitus may undergo natural recovery, but if improvement is not observed, surgical treatments such as resurfacing the vascular area with bone grafting can be effective [45-48].

Intratympanic injection: Effective placebo effect

Another approach involves intratympanic dexamethasone injection (ITDI). While some literature suggests its efficacy, a systematic review and meta-analysis revealed no significant effect compared to a placebo [49]. However, it is crucial to reconsider that there is no absolute absence of tinnitus reduction but rather a similar occurrence in both the group receiving ITDI and the group receiving saline solution. Among four randomized controlled trials (RCTs), one reported a significant effect compared to placebo [50], while the remaining three reported some reduction in tinnitus after ITDI, with a similar effect observed in the control groups [51-53].

The placebo effect associated with intratympanic injection does not seem exclusive to dexamethasone. In a clinical trial exploring the effects of intratympanic injection of an N-methyl-D-aspartate (NMDA) receptor antagonist on unilateral tinnitus, both the experimental and control groups showed a change of more than 10 points in the Tinnitus Functional Index (TFI) scores [54]. Ultimately, the act of injecting medication into the middle ear may induce a perceived therapeutic effect among patients. Although the conclusion is that there is no significant effect compared to placebo, from the patient's perspective, any subjective reduction in tinnitus could be perceived positively. If patients feel that their tinnitus has decreased even slightly through intratympanic injection, this perception may contribute to positive effects in conjunction with tinnitus counseling. Therefore, the placebo effect does not necessarily diminish the value of this treatment modality.

Rather than in refractory tinnitus cases, the efficacy of intratympanic steroid injections appears to be most favorable in cases of acute unilateral tinnitus [55]. The presence of a placebo effect is similarly noted [53]. However, in this scenario, it may be considered a specific type of sudden hearing loss with a relatively small degree of hearing loss. This aspect will be further explored in the following paragraph.

Tinnitus in sudden sensorineural hearing loss

Tinnitus can be a chief complaint in cases of sudden sensorineural hearing loss (SSNHL) with relatively small degree of hearing loss. SSNHL is defined as a rapid onset of hearing loss of 30 dB or more in three consecutive frequencies within 72 hours [56]. Although tinnitus commonly accompanies the substantial hearing loss of 30 dB or more, most cases seeking medical attention primarily complain of hearing impairment. However, in instances where the degree of hearing loss is smaller than this criterion or if the loss occurs only in three or fewer frequencies, patients may present with tinnitus rather than emphasizing hearing impairment [57,58]. Tinnitus can also be the primary complaint even in low-frequency limited losses [59]. It is essential to consider the possibility that tinnitus resulting from SSNHL may improve or even disappear when the hearing loss is restored. Through detailed medical history, determining when tinnitus occurred and promptly considering steroid therapy, especially if unilateral tinnitus suddenly develops, can be effective by considering the possibility of SSNHL.

Various other pharmacological treatments

Studies investigating gabapentin, a drug similar to carbamazepine, have been conducted. Although the placebo effect was observed, it was noted to have a relatively better response in tinnitus resulting from acoustic trauma [60,61]. Research on herbal medicines is also extensive. Medications containing Ginkgo biloba, known for their safety, are frequently prescribed to tinnitus patients. While their safety is considered certain, the evidence for their efficacy remains unclear [62]. Due to their superior safety, investigations explore the possibility of combination therapy with other formulations. Studies have looked into combining antioxidant vitamins A, C, E, selenium [63], and complex formulations including G. biloba along with magnesium, melatonin, vitamin B, and zinc. Significant improvements in tinnitus have been reported in some cases [64]. However, these are all single studies, and further research is needed to confirm their effectiveness. Another herbal preparation, St. John's wort (known for its antidepressant effects), combined with G. biloba, was studied in an RCT, but no superiority over monotherapy was concluded [65]. Ongoing interest in herbs persists, with research on complex formulations made from components such as Rosa canina, Urtica dioica, and Tanacetum vulgare, known to be effective against peripheral neuropathy symptoms, reporting a significant reduction in tinnitus compared to the control group [66]. While more research is needed to establish clearer results regarding efficacy, the promising aspect lies in their overall safety. The summary of the mentioned medications and their effects is outlined in Table 1.

**Table 1:** Summary of the effects and uses of medications for tinnitus. ITDI: intratympanic dexamethasone injection; SSRI: selective serotonin reuptake inhibitor; SSNHL: sudden sensorineural hearing loss

Types	Summary of the effectiveness
Antidepressants	Reducing overall tinnitus burden by controlling comorbid depressive symptoms in chronic subjective tinnitus. - SSRIs are the most recommended for safety.
Anxiolytics	Reducing tinnitus burden by controlling accompanying anxiety. - Be aware of the risk of abuse. - Clonazepam is the most recommended.
Carbamazepine	Effective in typewriter tinnitus (staccato or popping corn sound). - Used at 200-400 mg per day. - If effective, tinnitus reduction response within 2 weeks. - If no response, consider other types, such as middle ear myoclonus.
ITDI	It seems to work best in acute unilateral tinnitus. - May also experience a reduction in chronic tinnitus; this may be a placebo effect.
Steroids	In SSNHL, which is with minimal hearing loss.
Others	Gabapentin may be most responsive in tinnitus associated with acoustic trauma. - *Ginkgo biloba* is widely used because of its safety, but there is no clear evidence of its effectiveness. Combination therapy with other supplements (antioxidants, vitamins, other herbs) is being investigated.

## Conclusions

It is evident that counseling-based TRT and CBT are pivotal treatment for chronic subjective tinnitus. However, the judicious use of medications, especially those targeting comorbid depression or anxiety symptoms with antidepressants or anxiolytics, can contribute to alleviating the overall distress caused by tinnitus. Additionally, even if it is a placebo effect, subjective improvement in tinnitus symptoms can be experienced through intratympanic injections. Patients who undergo such experiences of tinnitus modulation may respond more effectively to TRT and CBT. Certain forms of tinnitus respond well to specific medications. Carbamazepine is effective in typewriter tinnitus, where patients perceive staccato sounds or popping corn-like noises. Intratympanic injections show the best response in acute unilateral tinnitus. In cases of SSNHL with minimal hearing loss, steroid treatment may not only restore hearing but also eliminate accompanying tinnitus.
